# UK prevalence of underlying conditions which increase the risk of severe COVID-19 disease: a point prevalence study using electronic health records

**DOI:** 10.1186/s12889-021-10427-2

**Published:** 2021-03-11

**Authors:** Jemma L. Walker, Daniel J. Grint, Helen Strongman, Rosalind M. Eggo, Maria Peppa, Caroline Minassian, Kathryn E. Mansfield, Christopher T. Rentsch, Ian J. Douglas, Rohini Mathur, Angel Y. S. Wong, Jennifer K. Quint, Nick Andrews, Jamie Lopez Bernal, J. Anthony Scott, Mary Ramsay, Liam Smeeth, Helen I. McDonald

**Affiliations:** 1 NIHR Health Protection Research Unit (HPRU) in Immunisation, London, UK; 2 London School of Hygiene & Tropical Medicine, Keppel Street, London, WC1E 7HT UK; 3 Public Health England, 61 Colindale Ave, London, NW9 5EQ UK; 4 Imperial College London, South Kensington, London, SW7 2BU UK

**Keywords:** Prevalence, Risk factors, COVID-19, Electronic health records, United Kingdom

## Abstract

**Background:**

Characterising the size and distribution of the population at risk of severe COVID-19 is vital for effective policy and planning. Older age, and underlying health conditions, are associated with higher risk of death from COVID-19. This study aimed to describe the population at risk of severe COVID-19 due to underlying health conditions across the United Kingdom.

**Methods:**

We used anonymised electronic health records from the Clinical Practice Research Datalink GOLD to estimate the point prevalence on 5 March 2019 of the at-risk population following national guidance. Prevalence for any risk condition and for each individual condition is given overall and stratified by age and region with binomial exact confidence intervals. We repeated the analysis on 5 March 2014 for full regional representation and to describe prevalence of underlying health conditions in pregnancy. We additionally described the population of cancer survivors, and assessed the value of linked secondary care records for ascertaining COVID-19 at-risk status.

**Results:**

On 5 March 2019, 24.4% of the UK population were at risk due to a record of at least one underlying health condition, including 8.3% of school-aged children, 19.6% of working-aged adults, and 66.2% of individuals aged 70 years or more. 7.1% of the population had multimorbidity. The size of the at-risk population was stable over time comparing 2014 to 2019, despite increases in chronic liver disease and diabetes and decreases in chronic kidney disease and current asthma. Separately, 1.6% of the population had a new diagnosis of cancer in the past 5 y.

**Conclusions:**

The population at risk of severe COVID-19 (defined as either aged ≥70 years, or younger with an underlying health condition) comprises 18.5 million individuals in the UK, including a considerable proportion of school-aged and working-aged individuals. Our national estimates broadly support the use of Global Burden of Disease modelled estimates in other countries. We provide age- and region- stratified prevalence for each condition to support effective modelling of public health interventions and planning of vaccine resource allocation. The high prevalence of health conditions among older age groups suggests that age-targeted vaccination strategies may efficiently target individuals at higher risk of severe COVID-19.

**Supplementary Information:**

The online version contains supplementary material available at 10.1186/s12889-021-10427-2.

## Background

The burden of mortality from COVID-19 rises steeply with age [1]. This is due to a combination of age itself, and the prevalence of underlying health conditions. Both age and underlying health conditions are independently associated with severe COVID-19 outcomes, including hospitalisation and mortality [2–5]. Many of the relevant underlying health conditions are also more common at older ages, and people with underlying health conditions account for the majority of COVID-19-related hospital and intensive care admissions [6]. This is reflected in United Kingdom (UK) national guidance describing individuals at moderate or high risk of severe COVID-19 (Table 1), which is based on age and underlying health conditions [7].

**Table 1:** COVID-19 moderate- and high-risk groups in national guidance [7] compared to the at-risk population study definition

COVID-19 moderate- and high-risk population definitions in national guidance for England [7]	Study definition
Anyone aged 70 years or older (regardless of medical conditions) is defined as at moderate risk.	The study definition of the at-risk population for all ages comprised individuals with any of the underlying health conditions in national guidance, incorporating high risk groups as indicated. Detailed inclusion criteria for each condition were based on national guidance defining influenza clinical risk groups, to reflect risk of respiratory infection [8].
Among individuals aged less than 70 years, people at moderate risk from coronavirus infection include people who: • have a lung condition that’s not severe (such as asthma, chronic obstructive pulmonary disease, emphysema or bronchitis) o *high risk* have been told by a doctor they have a severe lung condition (such as cystic fibrosis, severe asthma or severe chronic obstructive pulmonary disease) • have heart disease (such as heart failure) • have diabetes • have chronic kidney disease • have liver disease (such as hepatitis) • have a condition affecting the brain or nerves (such as Parkinson’s disease, motor neurone disease, multiple sclerosis or cerebral palsy) • have a condition that means they have a high risk of getting infections o *high risk* have a condition that means they have a very high risk of getting infections (such as SCID or sickle cell) o *high risk* have blood or bone marrow cancer (such as leukaemia, lymphoma or myeloma) • are taking medicine that can affect the immune system (such as low doses of steroids) o *high risk* are taking medicine that makes them much more likely to get infections (such as high doses of steroids or immunosuppressant medicine) o *high risk* have had an organ transplant o *high risk* are having chemotherapy or antibody treatment for cancer, including immunotherapy o *high risk* are having an intense course of radiotherapy (radical radiotherapy) for lung cancer o *high risk* are having targeted cancer treatments that can affect the immune system (such as protein kinase inhibitors or PARP inhibitors) o *high risk* have had a bone marrow or stem cell transplant in the past 6 months, or are still taking immunosuppressant medicine • are very obese (a BMI of 40 or above)	
• are pregnant o *high risk* have a serious heart condition and are pregnant	The prevalence of pregnancy was estimated separately from the general at-risk population.

Characterising the size and distribution of the population at risk of severe COVID-19 is vital for effective policy and planning in response to the COVID-19 pandemic [9]. Age- and region-specific prevalence of at-risk groups are key to predicting mortality and managing pressure on hospital inpatient and intensive care services across the country. Numbers of school-aged children and working-aged adults at risk are important for re-opening local schools and workplaces. Current international and UK national guidance advise that individuals at high risk of death from COVID-19 due to age or underlying health conditions should be a priority for COVID-19 vaccination [10, 11]. Vaccination planning requires at-risk population size for vaccine numbers, and age and regional distribution for modelling impact on regional transmission, since vaccine response typically decreases with older age [12].

Worldwide, modelling based on the Global Burden of Disease (GBD) study suggests that approximately one in five individuals have a health condition that increases risk of COVID-19 [13]. National prevalence studies of COVID-19 at-risk groups are rare. Large household surveys suggest that a third of adults in the United States, and between a third and a half of adults in Brazil, have at least one risk factor for COVID-19 (based on age ≥ 65 years, or underlying health conditions for younger adults) [14, 15]. A previous study estimated that at least 8.4 million individuals in the UK were at risk, but included only a subset of relevant health conditions [16]. Universal healthcare with an electronic health records system offers an opportunity for precise and representative estimation of at-risk prevalence in the UK, which may both support UK policy-making and offer a comparison with national GBD-based modelling estimates to aid in interpretation of GBD-based estimates internationally.

This study aimed to quantify the size, composition, and distribution of the population at risk of severe COVID-19 across the UK in March 2019, using electronic health records to define at-risk status based on all underlying conditions in UK national guidance.

## Methods

### Data sources

We conducted a point prevalence study among the UK general population using the Clinical Practice Research Datalink (CPRD) GOLD dataset, an anonymised sample of electronic health records from primary care practices across the UK [17]. The dataset includes diagnoses recorded using Read codes, primary care prescribing, and results of tests ordered in primary care. Data validity has been shown to be high [18]. The UK has universal healthcare, and the sample of the population who are in CPRD GOLD was found to be nationally representative by age and sex in March 2011: we re-assessed representativeness in 2019 in a sensitivity analysis [17].

Secondary care (hospital) data linkage is available for approximately 75% of CPRD GOLD-registered individuals in England, based on practice-level consent. For patients admitted to hospital, the Hospital Episode Statistics Admitted Patient Care dataset records diagnoses using International Classification of Diseases ICD-10 codes, and procedures such as chemotherapy using Classification of Interventions and Procedures OPCS-4 codes [19].

The CPRD Pregnancy Register uses validated algorithms, combining information across the primary care record such as antenatal scans, expected delivery dates, and deliveries, terminations and miscarriage records, to date and characterise pregnancies in CPRD GOLD [20].

### Index dates

Our primary analysis index date was 5 March 2019 for up-to-date national prevalence estimates. CPRD GOLD coverage peaked in 2014, when it included approximately 7% of the UK population: by 2019 the dataset was smaller and did not cover all regions in England. Since the dataset in 2014 therefore offered greater power than 2019, and full regional representation across England, we repeated point prevalence estimates for 5 March 2014 as a sensitivity analysis.

Pregnancy was described for the index date of 5 March 2014 only, not 5 March 2019, since the latest Pregnancy Register update was in February 2018.

### Study population

The study population comprised individuals aged 2–100 years with a current registration and a record meeting CPRD quality criteria (acceptable patient record and practice up to standard) in CPRD GOLD, with at least 1 y’s prior registration to allow recording of underlying conditions [21]. Eligibility started on the latest of: 1 January 2019, second birthday, a year after registration, or practice meeting CPRD quality standards. Eligibility ended at the earliest of: 5 March 2019, hundredth birthday, death, leaving the practice, or last data collection from the practice. Individuals with any time eligible between 1 January and 5 March were included in the main analysis of point prevalence on 5 March to increase study power, with a sensitivity analysis limited to individuals active in the dataset on 5 March 2019.

For pregnancy, the study population comprised women aged 11–49 years. As pregnancy is transient, women were required to be registered in the dataset on 5 March 2014, rather than any time between 1 January and 5 March 2014.

### Definition of at-risk population

In national guidance, all individuals aged ≥70 years are considered at moderate risk (Table 1) [7]. Since age-specific population estimates are readily available, the primary analysis for this study defined at-risk status based on underlying health conditions alone, rather than age. An additional analysis estimated the size of the at-risk population including all individuals aged ≥70 years.

We defined the COVID-19 at-risk population as individuals with *at least one* underlying health condition conferring moderate or high risk of severe COVID-19 according to national guidance (Table 1). Namely: any history of chronic respiratory disease (excluding asthma), heart disease, kidney disease, neurological conditions such as multiple sclerosis, diabetes mellitus; or current asthma, severe obesity, or immunosuppression; assessed on the index date [7].

Underlying conditions were defined using diagnoses, height and weight measurements, test results, and prescriptions recorded in primary care for the main analysis. Pregnancy status was ascertained from the CPRD Pregnancy Register (**Supplementary Table 1,** Additional File 1). Individuals with no recorded body mass index were included in the analysis, categorised as having no evidence of severe obesity. For analysis using linked secondary care data, diagnoses and procedures recorded in secondary care were additionally ascertained from ICD-10 and OCPS-4 codes respectively.

Multimorbidity was defined as more than one condition among the following domains: asthma or other chronic respiratory disease; chronic heart disease; chronic kidney disease; chronic liver disease; chronic neurological disease; diabetes; or immunosuppression (including individuals with dysplenia and organ transplant recipients).

Cancer survivors have an increased risk of COVID-19 mortality but non-haematological cancer survivors are only included in current COVID-19 guidance if receiving immunosuppressing treatment (Table 1) [2]. Separately to the study at-risk definition we described prevalence of any new cancer diagnosis in the past one and five years, as cancer survivors may be at increased risk of COVID-19 related death [2].

### Statistical analysis

Point prevalence estimates of the at-risk population and each underlying condition on 5 March 2019 were calculated per 100,000 with binomial exact 95% confidence intervals, for each nation in the UK. The at-risk population prevalence was stratified by sex and age, categorised in 5-year bands except 2–9 years and 90–99 years. Prevalence estimates for the at-risk population and each condition were stratified by age and region, separately and in combination. Prevalence values with fewer than five individuals were suppressed to preserve confidentiality.

For additional analysis estimating the size of the at-risk population including all individuals aged ≥70 years, the at-risk prevalence among individuals aged 2–69 years was age-standardised in 5-year bands, and added to the population aged ≥70 years, using mid-2019 national population estimates [22]. Comparison of prevalence in 2014 to 2019 was stratified by region to account for the change in regional representation of the dataset over time. The point prevalence of pregnancy and underlying health conditions was estimated among women aged 11–49 years on 5 March 2014. Prevalence estimates with and without linked secondary care records were compared among individuals at practices in England which had consented to data linkage.

#### Sensitivity analyses

CPRD GOLD was nationally representative by age and sex in March 2011 [17]. To update this assessment, the 2019 study population was compared to mid-2019 national population estimates, and 2019 at-risk prevalence estimates directly age-standardised in five-year bands using mid-2019 population estimates for each nation [22].

The main analysis included individuals eligible for any period of time between 1 January and 5 March 2019. Individuals who left CPRD between 1 January and 5 March would not subsequently have had new diagnoses recorded, which could underestimate point prevalence on 5 March. As a sensitivity analysis, at-risk prevalence was estimated with the study population restricted to individuals who were still registered in CPRD on 5 March 2019.

All analysis was conducted using STATA 16 MP.

## Results

### Characteristics of the study population

**Fig. 1:**
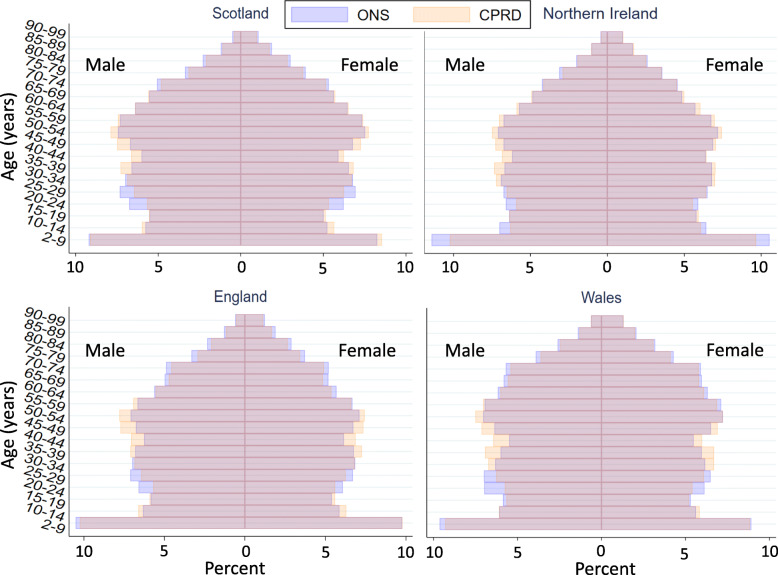
Age and sex distribution of 2019 study population (*N* = 2,706,053) compared to mid-2019 national population estimates [22]

The 2019 study population included 2,706,053 individuals: 990,939 (36.6%) in England, 801,352 in Scotland, 708,670 in Wales and 205,092 in Northern Ireland (Table 2). Approximately half (50.2%) were female. The study included 359,412 individuals (13.3%) aged ≥70 years. There was some over-representation of 40–59-year-olds compared to mid-2019 national population estimates for all four countries **(**Fig. 1**)**.

**Table 2:** Point prevalence^a^ of the COVID-19 at-risk population in the UK on 5 March 2019, *N* = 2,706,053

		Scotland		Northern Ireland		Wales		England	
		***N*** = 801,352		***N*** = 205,092		***N*** = 708,670		***N*** = 990,939	
		***n***	**Point prevalence /100,000 (95% CI)**	***n***	**Point prevalence /100,000 (95% CI)**	***n***	**Point prevalence /100,000 (95% CI)**	***n***	**Point prevalence /100,000 (95% CI)**
**Demographics**	Mean age (SD)		42.3 (22.6)		40.7 (22.7)		42.6 (23.2)		40.8 (22.8)
	Female	403,151	50,309 (50,199–50,418)	102,545	50,000 (49,783–50,216)	356,080	50,246 (50,130–50,363)	496,988	50,153 (50,055–50,252)
	Ethnicity								
	White	427,954	53,404 (53,295–53,513)	55,293	26,960 (26,768–27,153)	226,425	31,951 (31,842–32,059)	570,706	57,592 (57,495–57,690)
	South Asian	9325	1164 (1140–1187)	764	373 (347–400)	7440	1050 (1026–1074)	48,334	4878 (4835–4920)
	Black	3989	498 (482–513)	335	163 (146–182)	2861	404 (389–419)	27,947	2820 (2788–2853)
	Other	9360	1168 (1145–1192)	1102	537 (506–570)	4497	635 (616–653)	20,106	2029 (2001–2057)
	Mixed	2323	290 (278–302)	253	123 (109–140)	2219	313 (300–326)	12,376	1249 (1227–1271)
	Not recorded	348,401	43,477 (43,368–43,585)	147,345	71,843 (71,648–72,038)	465,228	65,648 (65,537–65,759)	311,470	31,432 (31,340–31,523)
**Underlying health conditions contributing to the at-risk population**	Chronic liver disease	4125	515 (499–531)	681	332 (308–358)	2150	303 (291–316)	2525	255 (245–265)
	Chronic heart disease	39,804	4967 (4920–5015)	9740	4749 (4657–4842)	34,459	4862 (4813–4913)	37,040	3738 (3701–3775)
	Chronic respiratory disease (other than asthma)	28,529	3560 (3520–3601)	7846	3826 (3743–3910)	24,192	3414 (3372–3456)	26,821	2707 (2675–2739)
	Current asthma (only)	47,164	5886 (5834–5937)	14,636	7136 (7025–7249)	51,783	7307 (7247–7368)	61,762	6233 (6185–6280)
	Chronic neurological disease	29,631	3698 (3656–3739)	6729	3281 (3204–3359)	23,850	3365 (3324–3408)	27,488	2774 (2742–2806)
	Diabetes mellitus	54,987	6862 (6807–6917)	15,878	7742 (7627–7858)	59,995	8466 (8401–8531)	61,925	6249 (6202–6297)
	Organ transplant recipient	748	93 (87–100)	182	89 (76–103)	653	92 (85–99)	894	90 (84–96)
	Asplenia/sickle cell disease	1062	133 (125–141)	244	119 (105–135)	905	128 (120–136)	1337	135 (128–142)
	Other immunosuppression	7199	898 (878–919)	1616	788 (750–827)	4181	590 (572–608)	6419	648 (632–664)
	Chronic kidney disease	54,845	6844 (6789–6900)	17,296	8433 (8313–8554)	53,280	7518 (7457–7580)	69,894	7053 (7003–7104)
	Severe obesity (BMI ≥40 kg/m^2^)	21,249	3315 (3271–3359)	5256	3299 (3211–3387)	21,431	3803 (3753–3853)	21,282	2762 (2725–2799)
**At-risk population based on underlying health conditions**	At-risk population overall^b^	196,378	24,506 (24,412–24,600)	53,945	26,303 (26,112–26,494)	187,695	26,486 (26,383–26,588)	223,587	22,563 (22,481–22,646)
	among those aged < 70 years	126,695 /696,563	18,189 (18,098–18,279)	36,178 /180,643	20,027 (19,843–20,213)	117,628 /603,155	19,502 (19,402–19,602)	143,305 /866,280	16,543 (16,464–16,621)
	among those aged ≥70 years	69,683 /104,789	66,498 (66,212–66,784)	17,767 /24,449	72,670 (72,106–73,228)	70,067 /105,515	66,405 (66,119–66,690)	80,282 /124,659	64,401 (64,135–64,667)
	of school age (5–18 years)	9680 /127,199	7610 (7465–7757)	3737 /35,846	10,425 (10,111–10,746)	10,105 /115,258	8767 (8605–8932)	13,915 /175,447	7931 (7805–8059)
	of working age (19–65 years)	99,786 /508,725	19,615 (19,506–19,724)	27,944 /129,274	21,616 (21,392–21,842)	91,644 /433,284	21,151 (21,030–21,273)	111,409 /618,404	18,016 (17,920–18,112)
	among males	93,319 /398,201	23,435 (23,304–23,567)	25,691 /102,534	25,056 (24,791–25,323)	89,178 /352,556	25,295 (25,151–25,439)	106,989 /493,908	21,662 (21,547–21,777)
	among females	103,059 /403,151	25,563 (25,429–25,698)	28,251 /102,545	27,550 (27,277–27,824)	98,513 /356,080	27,666 (27,519–27,813)	116,594 /496,988	23,460 (23,342–23,578)
**Multimorbidity**		58,076	7247 (7191–7304)	16,248	7922 (7806–8040)	55,556	7839 (7777–7902)	60,994	6155 (6108–6203)
**Cancer survivors**	Incident cancer in previous year	3439	429 (415–444)	899	438 (410–468)	3164	446 (431–462)	3742	376 (364–388)
	Incident cancer in previous 5 years	12,847	1603 (1576–1631)	3344	1630 (1576–1686)	12,451	1757 (1726–1788)	14,663	1480 (1456–1504)

*SD* standard deviation, *95% CI* 95% confidence interval, *BMI* body mass index

^a^Point prevalence estimates using standalone primary care records

^b^The at-risk population comprised individuals with: any history of chronic respiratory disease other than asthma, heart disease, kidney disease, neurological conditions such as multiple sclerosis, diabetes mellitus; or with current asthma, severe obesity, or immunosuppression; assessed at each index date.

In 2014, the dataset included 4,730,254 individuals: 2,980,402 (63.0%) in England, 810,169 (17.1%) in Scotland, 730,563 (15.4%) in Wales and 209,120 (4.4%) in Northern Ireland (**Supplementary Table 2,** Additional File 1). Age and sex distributions were similar to 2019, with 50.3% female and 12.7% aged ≥70 years.

### COVID-19 at-risk population

On 5 March 2019, 24.4% (95% CI 24.4–24.5) of the study population were at risk of severe COVID-19 due to underlying health conditions. National at-risk prevalence ranged from 22.6% in England to 26.5% in Wales (Table 2).

In a secondary analysis, the number of at-risk individuals based on current guidance including all individuals aged ≥70 years was estimated at 18.5 million across the UK, of whom 9.53 million (95% CI 9.52–9.53) were aged < 70 years (Table 3).

**Table 3:** Estimated size of the 2019 UK at-risk population according to national guidance (either aged ≥70 years, or younger with an underlying health condition)

	Age 2 to 69 years			Age ≥ 70 years	Estimated total number of individuals in the at-risk population
	Age-standardised prevalence of at least one underlying health condition /100,000 (95% CI)	Office for National Statistics mid-2019 population estimate	Estimated number of individuals aged 2–69 years with at least one underlying health condition (95% CI)	Office for National Statistics mid-2019 population estimate	
**Scotland**	18,046 (17,956–18,137)	4,615,093	832,848 (828,694–837,019)	744,701	1,577,549
**Northern Ireland**	19,672 (19,490–19,855)	1,622,687	319,213 (316,266–322,182)	224,851	544,064
**Wales**	19,519 (19,418–19,619)	2,609,759	509,389 (506,776–512,011)	480,234	989,623
**England**	16,573 (16,495–16,652)	47,467,071	7,866,870 (7,829,660–7,904,217)	7,556,976	15,423,846

*95% CI* 95% confidence interval.

#### Composition by underlying health conditions

The commonest conditions across the UK were chronic kidney disease (7.2%), diabetes mellitus (7.1%), asthma (6.5%) and chronic heart disease (4.5%). Prevalence of each condition varied nationally, with chronic liver disease notably commoner in Scotland (Table 2). Multimorbidity was common, ranging from 6.2% in England to 7.9% in Northern Ireland: 7.1% across the UK.

#### Variation by age

**Fig. 2:**
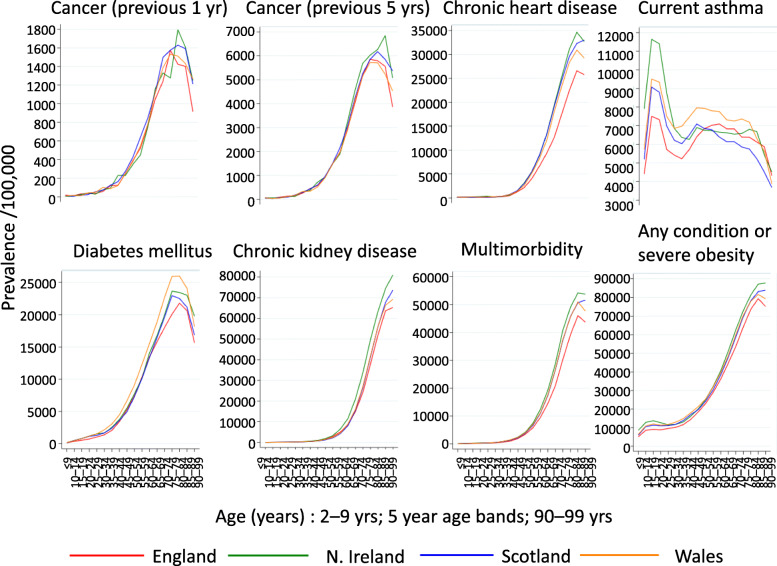
Age distributions of the at-risk population and underlying health conditions on 5 March 2019, *N* = 2,706,053

The proportion of at-risk individuals increased gradually with age from 5.1% of children aged 2–9 years to a peak at 79.4% of those aged 85–89 years in England before declining at older ages (Fig. 2). Similar age distributions were seen in each nation, and for each condition except current asthma, which peaked at age 10–14 years (Fig. 2).

The at-risk population comprised 18.1% of individuals aged < 70 years (including 8.3% of school-aged children and 19.6% of working aged adults) and 66.2% of individuals aged ≥70 years across the UK (Table 2).

#### Variation by sex

**Fig. 3:**
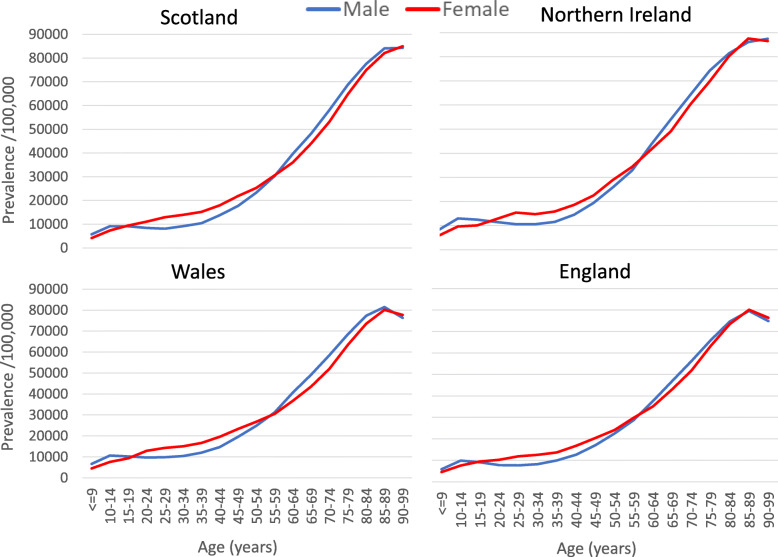
2019 point prevalence of the at-risk population by age and sex across the UK, *N* = 2,706,053

Overall, a higher proportion of women than men were at risk (Table 2), but the association varied with age, and men were more likely than women to be at risk from age 55 years upwards (Fig. 3**; Supplementary Table 3,** Additional File 1).

#### Variation by region

**Fig. 4:**
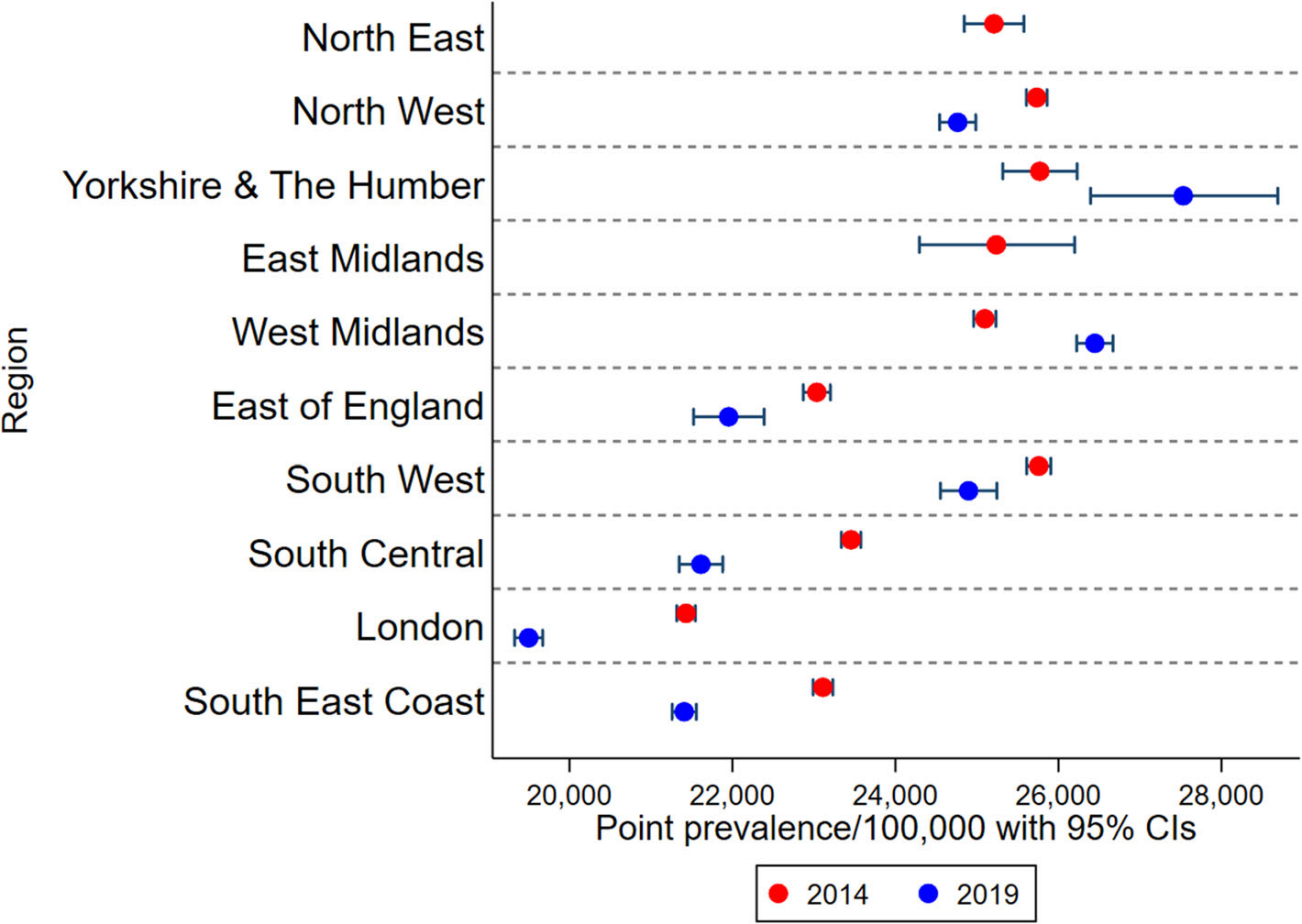
Point prevalence of the England at-risk population by region comparing 2014 (*N* = 4,730,254) and 2019 (*N* = 2,706,053). The 2019 study population did not include any individuals in the North East or East Midlands regions; x-axis scale starts at 20,000/100,000

No individuals from the North East or East Midlands regions of England were included in 2019, whereas all regions were represented in 2014. London had the lowest proportion of the population considered at risk in both 2014 and 2019 (Fig. 4). The East of England, South Central and South East also had lower prevalence of at-risk individuals than Midlands or Northern regions in both 2014 and 2019. Regional patterns varied between underlying conditions (**Supplementary Fig. 1,** Additional File 1).

Prevalence estimates of the at-risk population and each condition stratified by age and region (separately and combined) on 5 March 2014 and 2019 are here: 10.17037/DATA.00001833

#### Differences between 2014 and 2019 prevalence estimates

Compared to 2014, at-risk prevalence estimates in 2019 were 0.8% higher in Northern Ireland but lower in Scotland (− 0.5%), Wales (− 0.8%), and England (− 1.3%). When stratified by region within England (Fig. 4), at-risk prevalence increased from 2014 to 2019 for Yorkshire and the Humber and the West Midlands, and decreased in all other regions (excluding the North East and East Midlands, which were unavailable in the 2019 dataset), but no changes exceeded 1.9% difference.

For underlying conditions, absolute changes in UK prevalence estimates from 2014 to 2019 ranged from a − 0.9% decrease in chronic kidney disease to a 0.7% increase in diabetes mellitus (**Supplementary Fig. 1,** Additional File 1). The biggest relative increases were for chronic liver disease (+ 32.3% from 2014 to 2019), diabetes (+ 11.5%) and chronic respiratory disease other than asthma (+ 11.4%). The largest relative falls were for chronic kidney disease (− 10.6%) and current asthma (− 6.0%).

#### Cancer survivors

On 5 March 2019, 0.4% of the UK had incident cancer recorded within the previous year and 1.6% within the previous 5 y (Table 2).

#### Pregnancy

Among women aged 11–49 years on 5 March 2014, 2.1% were pregnant, of whom 12.9% had a recorded health condition, compared to 14.5% of non-pregnant women (Table 4).

**Table 4:** 2014 prevalence^a^ of pregnancy and underlying health conditions among women aged 11–49 years, *N* = 1,181,840

	Scotland ***N*** = 205,542		Northern Ireland ***N*** = 55,559		Wales ***N*** = 177,412		England ***N*** = 743,327	
Among all women aged 11–49 years	***n***	Point prevalence /100,000 (95% CI)	***n***	Point prevalence /100,000 (95% CI)	***n***	Point prevalence /100,000 (95% CI)	***n***	Point prevalence /100,000 (95% CI)
Pregnant or any underlying health condition^b^	34,051	16,566 (16,406–16,728)	9234	16,620 (16,311–16,932)	31,140	17,552 (17,376–17,730)	117,680	15,832 (15,749–15,915)
Any underlying health condition^b^	31,389	15,271 (15,116–15,428)	8317	14,970 (14,674–15,269)	28,733	16,196 (16,024–16,368)	102,121	13,738 (13,660–13,817)
Pregnant	3067	1492 (1440–1545)	1069	1924 (1811–2042)	2834	1597 (1540–1657)	17,755	2389 (2354–2424)
**Prevalence of underlying health conditions,** ^b^ **stratified by pregnancy status, among women aged 11–49 years**								
Pregnant with an underlying health condition^b^	405	13,205 (12,026–14,455)	152	14,219 (12,179–16,458)	427	15,067 (13,769–16,458)	2196	12,368 (11,887–12,862)
Not pregnant, with an underlying health condition^b^	30,984	15,303 (15,146–15,460)	8165	14,984 (14,686–15,287)	28,306	16,214 (16,041–16,388)	99,925	13,772 (13,693–13,851)

*95% CI* 95% confidence interval.

^a^ Point prevalence on 5 March 2014.

^b^ Underlying health conditions comprised: any history of chronic respiratory disease other than asthma, heart disease, kidney disease, neurological conditions such as multiple sclerosis, diabetes mellitus; or current asthma, severe obesity, or immunosuppression; assessed at each index date.

### Linked secondary care records

At-risk prevalence based on standalone primary care records was similar among individuals with and without eligibility for data linkage. Linked secondary care records increased the estimated prevalence of the at-risk population in England by 1.8% in both 2014 and 2019. The increase was greater among individuals < 70 years than those ≥70 years.

For underlying conditions, the greatest absolute changes in prevalence estimates were for multimorbidity, which increased from 6.5 to 7.6% in 2019, and chronic heart disease, which increased from 4.0 to 5.3%. The greatest relative increase was for chronic liver disease, which nearly doubled from 0.27 to 0.53% (Table 5).

**Table 5:** Prevalence estimates for England with and without linked secondary care data (2014 *N* = 1,802,468; 2019 *N* = 744,496)

	Individuals in England eligible for secondary care data linkage in 2014 ***N*** = 1,802,468				Individuals in England eligible for secondary care data linkage in 2019 ***N*** = 744,496			
	Standalone primary care data		Both primary and secondary care data		Standalone primary care data		Both primary and secondary care data	
	*n*	Point prevalence /100,000 (95% CI)	*N*	Point prevalence /100,000 (95% CI)	*n*	Point prevalence /100,000 (95% CI)	*n*	Point prevalence /100,000 (95% CI)
**Underlying health conditions contributing to the at-risk population**								
Chronic liver disease	4499	250 (242–257)	8104	450 (440–459)	2022	272 (260–284)	3941	529 (513–546)
Chronic heart disease	87,217	4839 (4807–4870)	108,711	6031 (5997–6066)	29,399	3949 (3905–3993)	39,083	5250 (5199–5300)
Chronic respiratory disease	53,358	2960 (2936–2985)	63,562	3526 (3500–3553)	21,345	2867 (2829–2905)	26,407	3547 (3505–3589)
Current asthma (only)	125,548	6965 (6928–7003)	136,702	7584 (7546–7623)	48,009	6449 (6393–6505)	53,030	7123 (7065–7182)
Chronic neurological disease	60,482	3356 (3329–3382)	69,932	3880 (3852–3908)	21,818	2931 (2892–2969)	26,284	3530 (3489–3573)
Diabetes mellitus	110,144	6111 (6076–6146)	114,149	6333 (6297–6369)	48,461	6509 (6453–6566)	50,760	6818 (6761–6876)
Organ transplant recipient	1470	82 (77–86)	1732	96 (92–101)	707	95 (88–102)	838	113 (105–120)
Asplenia/sickle cell disease	2545	141 (136–147)	2878	160 (154–166)	1089	146 (138–155)	1346	181 (171–191)
Other immunosuppression	14,134	784 (771–797)	32,537	1805 (1786–1825)	5035	676 (658–695)	7663	1029 (1006–1052)
Chronic kidney disease	159,241	8835 (8793–8876)	159,379	8842 (8801–8884)	54,661	7342 (7283–7401)	54,774	7357 (7298–7417)
Severe obesity (BMI ≥40 kg/m^2^)	36,668	2581 (2555–2607)	36,668	2581 (2555–2607)	16,706	2898 (2855–2942)	16,706	2898 (2855–2942)
**At-risk population**^a^								
At-risk population^a^	450,601	24,999 (24,936–25,062)	483,071	26,801 (26,736–26,865)	175,420	23,562 (23,466–23,659)	188,716	25,348 (25,249–25,447)
among those aged < 70 years	276,158	17,756 (17,696–17,816)	303,528	19,516 (19,453–19,578)	112,168	17,286 (17,194–17,378)	123,038	18,961 (18,866–19,057)
among those aged ≥70 years	174,443	70,579 (70,399–70,759)	179,543	72,643 (72,466–72,818)	63,252	66,157 (65,856–66,457)	65,678	68,694 (68,399–68,988)
Multimorbidity	133,803	7423 (7385–7462)	155,802	8644 (8603–8685)	48,009	6449 (6393–6505)	56,827	7633 (7573–7693)
**Cancer survivors**								
Incident cancer in previous year	8343	463 (453–473)	10,680	593 (581–604)	2936	394 (380–409)	3806	511 (495–528)
Incident cancer in previous 5 years	31,195	1731 (1712–1750)	36,815	2042 (2022–2063)	11,708	1573 (1544–1601)	13,854	1861 (1830–1892)

95% CI, 95% confidence interval.

^a^The at-risk population comprised individuals with: any history of chronic respiratory disease other than asthma, heart disease, kidney disease, neurological conditions such as multiple sclerosis, diabetes mellitus; or with current asthma, severe obesity, or immunosuppression; assessed at each index date.

### Sensitivity analyses

Age-standardisation did not alter 2019 at-risk prevalence estimates (not presented). When the study population was restricted to individuals active on 5 March 2019, at-risk prevalence estimates fell by less than 1% (**Supplementary Table 4,** Additional File 1).

## Discussion

This paper describes the size and distribution of the population at risk of severe COVID-19 based on clinical records from a large, nationally representative cohort across the UK.

On 5 March 2019, 24.4% of the UK population were at higher risk than others of the same age due to underlying health conditions, including 8.3% of school-aged children, 19.6% of working-aged adults, and 66.2% of individuals aged ≥70 years. The commonest conditions were chronic kidney disease, diabetes and asthma. Multimorbidity was common at 7.1%. The size and regional distribution of the at-risk population was similar in 2014 and 2019, with lower prevalence in London and the South of England than Midlands or Northern regions. Separately, the 1.6% of the study population with a new diagnosis of cancer within the previous 5 y may also be at increased risk of severe COVID-19 [2].

Including all individuals aged ≥70 years, 18.5 million individuals in the UK would be considered moderate or high risk under current national guidance [7]. This is higher than a previous estimate of 8.4 million comprising 7.2% of men and 7.5% of women aged 30–69 years, and 33% of men and 29% of women aged ≥70 years [16]. Our estimates include additional conditions to cover the full national guidance [7]. There were also differences in ascertainment: for example, our diabetes prevalence estimate for ages 30–69 in England was 7.0%, compared to 2.2% in the previous study [16] and 6.9% in the national Quality Outcomes Framework (QOF) [23]. This may be due to increases in diagnoses and recording of diabetes over time in our more recent study period of March 2019 (rather than 1997–2017) [16].

Our at-risk prevalence estimates were slightly lower than GBD-based estimates that 29.1% of the UK population had at least one underlying health condition increasing COVID-19 risk, or 28.1% when restricted to the same set of conditions by excluding cancers causing indirect immunosuppression and tuberculosis from GBD-based estimates [13]. This did not appear to be due to under-estimation of clustering due to multimorbidity in the GBD-based study, as the 9.2% multimorbidity prevalence modelled was higher than we observed even when using linked secondary care records. The difference was greatest among older age groups, and our finding that 19.6% of working-aged adults (19–65 years) were at risk is broadly comparable to the GBD-based estimate (for the same conditions) of 22.8% among those aged 15–64 years [13].

Our prevalence estimates are in line with national QOF diabetes and cancer monitoring, slightly higher than the more narrowly defined QOF chronic heart disease estimates [23], and consistent with previous UK studies of chronic kidney disease and asthma [24, 25]. The five-year trends of increasing diabetes and decreasing asthma prevalence are consistent with directions of change in a previous study of asthma [24], and QOF, although 2014 QOF diabetes prevalence was slightly higher at 6.2% in 2013/14 [23].

Linked secondary care records in England increased the estimated size of the at-risk population only modestly, but the estimated prevalence of chronic liver disease in 2019 nearly doubled from 0.27 to 0.53%, and multimorbidity and chronic heart disease prevalence also increased. Our chronic liver disease prevalence estimate in England of 529/100,000 when supplemented with secondary care data is more consistent with previous national estimates of approximately 600/100,000 for the UK than our lower estimate using primary care data alone [26]. Several studies of the associations between underlying health conditions and COVID-19 outcomes in England have used standalone primary care records to characterise underlying health conditions [3, 27]. Such studies may under-ascertain chronic liver disease, heart disease and multimorbidity, and thus underestimate associations of these conditions with COVID-19 outcomes. If the risk of severe COVID-19 differs between underlying health conditions, then their differential under-ascertainment in primary health records may bias estimates of associations of underlying health conditions with COVID-19 outcomes. Among women who were pregnant on 5 March 2014, 12.9% were at risk due to an underlying health condition, compared to a third of the pregnant women admitted to hospital with COVID-19 [28]. While the pregnancy register has high sensitivity for livebirths, pregnancy losses may be under-recorded [20]. The 2.1% point prevalence estimate of pregnancy is perhaps low compared to a survey in which 591/5686 (10%) of women aged 16–44 years in Britain reported a pregnancy ending in the previous year, although these are not easily comparable [29]. Caution is required in applying historical pregnancy estimates, as COVID-19 may affect family planning.

### Strengths and limitations

To our knowledge, these are the first prevalence estimates of the full population at risk of severe COVID-19 across the UK according to national guidelines. Strengths include the large, nationally representative cohort, risk group definitions with detailed ascertainment tailored to risk of COVID-19, and quantification of the value of linked secondary care records.

Our definition of the at-risk population was based on UK national guidance [7]. Large national studies have found that the health conditions in national COVID-19 guidance are indeed associated with increased risk of COVID-19-related death, although the size of associations vary [2–4]. Older individuals have also been found to be at higher risk of severe COVID-19 than younger, independent of underlying health conditions [2–4]. However, understanding of the risk factors for severe COVID-19 is still evolving. To support policy and planning to adapt flexibly to future evidence of the associations of different underlying conditions with COVID-19 outcomes, we provide age- and region- stratified prevalence for each underlying condition separately, including separating asthma from other respiratory conditions [27, 30].

A key limitation is that UK-wide estimates rely on primary care records, which may miss undiagnosed conditions and under-ascertain conditions diagnosed in secondary care. Our analysis including linked secondary care records in England suggests that estimates of the overall size of the at-risk population are robust, but that the prevalence of multimorbidity, chronic heart disease and liver disease may be underestimated from primary care records. There is likely under-ascertainment of immunosuppressing cancer treatments even using secondary care records, which could be on a scale similar to the 1.6% of the population newly diagnosed with cancer within the previous year. Second, the 2019 estimates did not include all regions in England. Although the dataset remained nationally representative in terms of age and sex in 2019, and prevalence estimates of individual conditions were consistent with expectations, suggesting that national 2019 estimates are representative, regionally-stratified estimates in 2019 are incomplete. Prevalence estimates from 2014 include all regions but are less up-to-date, and differences from 2019 may reflect changes in prevalence and recording of conditions, and the CPRD GOLD population over time. Third, inclusion of individuals active in the dataset at any point between 1 January and 5 March could have resulted in some under-estimation of point prevalence on 5 March: sensitivity analysis suggested this was minimal. Finally, we were able to describe pregnancy in 2014 only, and pregnancy prevalence may be under-estimated.

## Conclusions

We estimate that current national guidance on COVID-19 risk groups encompasses 18.5 million individuals across the UK, a larger population than previously estimated. These national estimates broadly support the use of Global Burden of Disease modelled estimates and age-targeted vaccination strategies in other countries.

We found that 66.2% of individuals aged ≥70 years had at least one recorded underlying condition, suggesting that an age-based approach to COVID-19 vaccination could efficiently target individuals at highest risk. Implementation of public health measures such as influenza vaccination generally achieve higher uptake when targeted on the basis of age rather than health conditions [31]. Age-based vaccination strategies may also be more feasible to implement in low-resource settings.

Our finding that 8.3% of school-aged children and 19.6% of working aged adults are in the at-risk population (as currently defined) emphasises the need to consider younger at-risk individuals in shielding guidance and when reopening schools and workplaces. The large number of children and younger adults with underlying conditions, who may nevertheless be at low absolute risk of severe COVID-19, supports vaccine strategies based on age- and condition-specific estimates of risk of severe COVID-19, rather than including individuals of any age with underlying conditions. We provide age-stratified prevalence for each condition to support effective vaccine resource allocation based on age and health conditions.

## Supplementary Information

**Additional file 1: Supplementary Table 1.** Study definitions of underlying health conditions and pregnancy. **Supplementary Table 2.** Study population characteristics and point prevalence of the COVID-19 at-risk population on 5 March 2014 in the United Kingdom using standalone primary care data, *N* = 4,730,254. **Supplementary Table 3.** The at-risk population prevalence on 5 March 2019 stratified by age and sex. **Supplementary Table 4.** At-risk prevalence estimates on 5 March 2019 with and without individuals who left CPRD between 1st January and 5th March 2019. **Supplementary Figure 1.** Point prevalence of the at-risk group and contributing underlying health conditions by region on 5 March 2014 compared to 5 March 2019 (2019 does not include any individuals in the North East or East Midlands regions; x-axis scales vary by condition).

## Data Availability

The data used for this study were obtained from the Clinical Practice Research Datalink (CPRD). All CPRD data are available via an application to the Independent Scientific Advisory Committee (see https://www.cprd.com/Data-access). Data acquisition is associated with a fee and data protection requirements. This manuscript is supported by shared codelists used to define each condition, and estimates of the prevalence of the at-risk population and each underlying health condition, stratified by age and region (separately and combined) are available at 10.17037/DATA.00001833.

## Abbreviations

UK: United Kingdom
GBD: Global Burden of Disease
CPRD GOLD: Clinical Practice Research Datalink GOLD
BMI: Body Mass Index
QOF: Quality Outcomes Framework
