# Supplementary material for: UK prevalence of underlying conditions which increase the risk of severe COVID-19 disease: a point prevalence study using electronic health records
Source: BMC Public Health. 2021 Mar 11;21:484. doi: 10.1186/s12889-021-10427-2 (PMC7948667; doi:10.1186/s12889-021-10427-2)

**ADDITIONAL FILE 1**

**Supplementary Table 1: Study definitions of underlying health conditions and pregnancy**

Detailed definitions of the underlying health conditions in the COVID-19 moderate and high risk groups were implemented according to national guidance on influenza and pneumococcal vaccination of clinical risk groups at risk of severe respiratory infection.

**Codelists:** We used validated ‘specific’ asthma Read codes published by Nissen et al.^27^ and cancer Read and ICD-10 codes published by Strongman et al. ^28^ The codelists and algorithms for the Pregnancy Register are described in Minassian et al. ^14^ Codelists for immunosuppression are available by Grint et al.^29^, and for neurological disease <https://doi.org/10.17037/DATA.00001704> and all other codelists are available at <https://datacompass.lshtm.ac.uk/1659/>

| **Underlying health condition** | **Study definition** | | **Included in study definition of the at-risk population** | **Moderate risk group in national guidance** | **High risk groups as defined in national guidance** |
| --- | --- | --- | --- | --- | --- |
| **Included in study definition of the at-risk population** | | | | | |
| Chronic liver disease | Any previous diagnosis of a chronic liver disease including cirrhosis, oesophageal varices, biliary atresia and chronic hepatitis. | | **✓** | **✓** |  |
| Chronic heart disease | Chronic heart disease likely to cause long-term increased risk of severe respiratory infection, including angina or myocardial infarction, heart disease, major congenital anomalies requiring long-term follow up such as Fallot’s tetralogy. | | **✓** | **✓** |  |
| Chronic respiratory disease | Any previous diagnosis of a chronic (long-term) respiratory disease, such as chronic obstructive pulmonary disease (COPD), emphysema or bronchitis, including cystic fibrosis and fibrosing interstitial lung diseases. | | **✓** | **✓** | People with a severe lung condition (such as cystic fibrosis or severe COPD). |
| Current asthma (only) | The age distribution of individuals with any history of an asthma diagnosis suggested misclassification of individuals with chronic obstructive pulmonary disease and childhood asthma, rather than current asthma.  Asthma as a qualifying condition to the clinically vulnerable population was therefore restricted to individuals with asthma diagnoses recorded within the previous three years. Individuals were described separately as having current asthma (only) if they had a current diagnosis of asthma and no diagnosis of chronic obstructive pulmonary disease (COPD). Individuals with co-diagnosis of asthma and COPD were included in the ‘chronic respiratory disease’ category. | | **✓** | **✓** | People with a severe lung condition such as severe asthma |
| Chronic neurological disease | Any previous diagnosis of stroke, transient ischaemic attack, or conditions in which respiratory function may be compromised due to neurological disease such as Parkinson’s disease, motor neurone disease, multiple sclerosis (MS). | | **✓** | **✓** |  |
| Diabetes mellitus | Any previous diagnosis of diabetes mellitus, or a diagnosis within the previous three months if a reversible cause was specified. | | **✓** | **✓** |  |
| Organ transplant recipients | Any previous history of any solid organ transplant (unless as donor) | | **✓** | **✓** | People who have had an organ transplant |
| Asplenia/sickle cell disease | Any previous history of asplenia or dysfunction of the spleen (including sickle cell disease but not sickle cell trait) | | **✓** | **✓** | People who have sickle cell disease. |
| Other immunosuppression | The previously published algorithm defining immunosuppression included permanent conditions (such as genetic conditions, HIV, and organ transplants), recent diagnoses and procedures (including haematological cancers, bone marrow transplants, and chemotherapy and radiotherapy if recorded), and medications including dose thresholds for oral steroids, methotrexate and azathioprine.^29^ | |  |  |  |
|  | **Based on diagnoses or procedures** | Any history of HIV or other permanent immunosuppression (such as genetic conditions compromising immune function) | **✓** | **✓** | People who: are having chemotherapy or antibody treatment for cancer, including immunotherapy; are having an intense course of radiotherapy (radical radiotherapy) for lung cancer; have a condition that means they have a very high risk of getting infections (such as SCID). |
|  |  | Any history in the previous year of: aplastic anaemia, leukaemia, lymphoma, receiving a bone marrow transplant, or receiving chemotherapy or radiotherapy. Any record within the previous six months of the end of a course of chemotherapy or radiotherapy. | **✓** | **✓** |  |
|  |  | Any record within the previous 3 months of a status of immunosuppression without further details. | **✓** | **✓** |  |
|  | **Based on medications** | Any prescription of biologic therapies within the previous year. | **✓** | **✓** | People who are taking medicine that makes them much more likely to get infections (such as high doses of steroids or immunosuppressant medicine) |
|  |  | Within the previous 3 months, any of:  short term high-dose corticosteroids >40mg prednisolone per day for more than 1 week;  or long term lower dose corticosteroids >20mg prednisolone per day for more than 14 day;  or Methotrexate >25mg per week; Azathioprine >3.0mg/kg/day; 6-mercaptopurine >1.5mg/kg/day;  or Corticosteroid injections; other DMARDS; other immunosuppressant medications. | **✓** | **✓** |  |
| Chronic kidney disease (CKD) | Any previous history of dialysis or renal transplant;  or a Read code recording CKD stage 3–5 or nephrotic syndrome without a more recent Read code recording CKD stage 1–2;  or latest estimated glomerular filtration rate (eGFR) ≤60 mL/min/1.73m^2^ using serum creatinine test results. | | **✓** | **✓** |  |
| Severe obesity | Latest body mass index on the index date ≥40 kg/m^2^, based on latest adult records of height and weight (18 years and above), and reported for age groups ≥20 years. | | **✓** | **✓** |  |
| **Study definition of multimorbidity within at-risk population** | | | | | |
| Multimorbidity | Multimorbidity was defined as more than one condition among the following domains: asthma or other chronic respiratory disease; chronic heart disease; chronic kidney disease; chronic liver disease; chronic neurological disease; diabetes; or immunosuppression (including individuals with dysplenia and organ transplant recipients). | | Subset of at-risk population | Subset of at-risk population |  |
| **Not included within study definition of at-risk population** | | | | | |
| Pregnancy | Any ongoing pregnancy episode on 5 March 2014 recorded in the CPRD pregnancy register (based on antenatal records, and not restricted by outcome of pregnancy). | | **No – described separately** | **✓** | People who have a serious heart condition and are pregnant. |
| Cancer | A new diagnosis of malignant cancer in the past year (no previously recorded diagnosis for that individual) or in the previous five years. | | **No – described separately** | No |  |

**Supplementary Table 2: Study population characteristics and point prevalence of the COVID-19 at-risk population on 5 March 2014 in the United Kingdom using standalone primary care data, N=4,730,254**

|  | **Scotland** | | **Northern Ireland** | | **Wales** | | **England** | |
| --- | --- | --- | --- | --- | --- | --- | --- | --- |
|  | **N=** **810,169** | | **N=** **209,120** | | **N=** **730,563** | | **N= 2,980,402** | |
| **Demographics** |  |  |  |  |  |  |  |  |
| Mean age (SD) |  | 41.5 (22.3) |  | 40.0 (22.4) |  | 42.4 (23.1) |  | 41.5 (22.9) |
|  | **n** | **Point prevalence /100,000 (95% CI)** | **n** | **Point prevalence /100,000 (95% CI)** | **n** | **Point prevalence /100,000 (95% CI)** | **n** | **Point prevalence /100,000 (95% CI)** |
| Female | 408,194 | 50,384 (50,275, 50,493) | 105,081 | 50,249 (50,035, 50,464) | 365,977 | 50,095 (49,980, 50,210) | 1,502,365 | 50,408 (50,351, 50,465) |
| Ethnicity |  |  |  |  |  |  |  |  |
| White | 416,913 | 51,460 (51,351, 51,569) | 44,561 | 21,309 (21,133, 21,485) | 197,002 | 26,966 (26,864, 27,068) | 1,535,724 | 51,527 (51,471, 51,584) |
| South Asian | 8,169 | 1,008 (987, 1,030) | 478 | 229 (209, 250) | 5,701 | 780 (760, 801) | 111,000 | 3,724 (3,703, 3,746) |
| Black | 3,344 | 413 (399, 427) | 207 | 99 (86, 113) | 1,923 | 263 (252, 275) | 71,218 | 2,390 (2,372, 2,407) |
| Other | 8,142 | 1,005 (983, 1,027) | 800 | 383 (357, 410) | 3,221 | 441 (426, 456) | 40,705 | 1,366 (1,353, 1,379) |
| Mixed | 1,529 | 189 (179, 198) | 166 | 79 (68, 92) | 1,443 | 198 (187, 208) | 25,443 | 854 (843, 864) |
| Not recorded | 372,072 | 45,925 (45,817, 46,034) | 162,908 | 77,902 (77,723, 78,079) | 521,273 | 71,352 (71,248, 71,456) | 1,196,312 | 40,139 (40,084, 40,195) |
| **Underlying health conditions contributing to ‘clinically vulnerable’ population** | | | | | | | | |
| Chronic liver disease | 3,485 | 430 (416, 445) | 555 | 265 (244, 288) | 1,768 | 242 (231, 254) | 6,715 | 225 (220, 231) |
| Chronic heart disease | 41,457 | 5,117 (5,069, 5,165) | 10,224 | 4,889 (4,797, 4,982) | 39,010 | 5,340 (5,288, 5,392) | 130,385 | 4,375 (4,352, 4,398) |
| Chronic respiratory disease | 26,665 | 3,291 (3,253, 3,330) | 6,785 | 3,245 (3,169, 3,321) | 23,718 | 3,247 (3,206, 3,287) | 79,935 | 2,682 (2,664, 2,700) |
| Current asthma (only) | 53,053 | 6,548 (6,495, 6,602) | 15,653 | 7,485 (7,373, 7,599) | 54,267 | 7,428 (7,368, 7,488) | 203,017 | 6,812 (6,783, 6,840) |
| Chronic neurological disease | 28,452 | 3,512 (3,472, 3,552) | 6,546 | 3,130 (3,056, 3,206) | 24,358 | 3,334 (3,293, 3,376) | 90,825 | 3,047 (3,028, 3,067) |
| Diabetes mellitus | 54,300 | 6,702 (6,648, 6,757) | 13,711 | 6,557 (6,451, 6,663) | 60,999 | 8,350 (8,286, 8,413) | 173,331 | 5,816 (5,789, 5,842) |
| Organ transplant recipient | 593 | 73 (67, 79) | 167 | 80 (68, 93) | 615 | 84 (78, 91) | 2,346 | 79 (76, 82) |
| Asplenia/sickle cell disease | 1,115 | 138 (130, 146) | 235 | 112 (98, 128) | 940 | 129 (121, 137) | 4,110 | 138 (134, 142) |
| Other immunosuppression | 6,790 | 838 (818, 858) | 1,525 | 729 (693, 767) | 4,830 | 661 (643, 680) | 22,054 | 740 (730, 750) |
| Chronic kidney disease | 58,614 | 7,235 (7,178, 7,291) | 17,601 | 8,417 (8,298, 8,537) | 61,631 | 8,436 (8,372, 8,500) | 244,216 | 8,194 (8,163, 8,225) |
| Severe obesity (BMI ≥40 kg/m^2^) | 21,076 | 3,269 (3,226, 3,313) | 4,725 | 2,919 (2,837, 3,002) | 21,135 | 3,634 (3,586, 3,682) | 61,041 | 2,609 (2,589, 2,630) |
| **At-risk population** | | | | | | | | |
| At-risk population^1^ | 202,815 | 25,034 (24,939, 25,128) | 53,270 | 25,473 (25,287, 25,661) | 198,033 | 27,107 (27,005, 27,209) | 712,737 | 23,914 (23,866, 23,963) |
| Multimorbidity^2^ | 59,198 | 7,307 (7,250, 7,364) | 15,609 | 7,464 (7,352, 7,578) | 60,234 | 8,245 (8,182, 8,308) | 200,752 | 6,736 (6,707, 6,764) |
| **Cancer** | | | | | | | | |
| Incident cancer in previous year | 3,485 | 430 (416, 445) | 768 | 367 (342, 394) | 3,145 | 430 (416, 446) | 12,281 | 412 (405, 419) |
| Incident cancer in previous 5 years | 12,721 | 1,570 (1,543, 1,597) | 3,170 | 1,516 (1,464, 1,569) | 11,720 | 1,604 (1,576, 1,633) | 47,412 | 1,591 (1,577, 1,605) |

SD, standard deviation; 95% CI, 95% confidence interval.

1. The at-risk population comprised individuals with: any history of chronic respiratory disease other than asthma, heart disease, kidney disease, neurological conditions such as multiple sclerosis, diabetes mellitus; or with current asthma, severe obesity, or immunosuppression; assessed at the index date of 5 March 2014.

2. Multimorbidity was defined as more than one condition among the following domains: asthma or other chronic respiratory disease; chronic heart disease; chronic kidney disease; chronic liver disease; chronic neurological disease; diabetes; or immunosuppression (including individuals with dysplenia and organ transplant recipients).

**Supplementary Table 3: The at-risk population prevalence on 5 March 2019 stratified by age and sex**

|  | **Scotland**  **N=801,352** | | | | **Northern Ireland**  **N=205,092** | | | | **Wales**  **N=708,670** | | | | **England**  **N=990,939** | | | |
| --- | --- | --- | --- | --- | --- | --- | --- | --- | --- | --- | --- | --- | --- | --- | --- | --- |
|  | **Male** | | **Female** | | **Male** | | **Female** | | **Male** | | **Female** | | **Male** | | Female | |
|  | **N=398,201** | | **N=403,151** | | **N=102,534** | | **N=102,545** | | **N=352,556** | | **N=356,080** | | **N=493,908** | | N=496,988 | |
|  | n | Point prevalence /100,000 (95% CI) | n | Point prevalence /100,000 (95% CI) | n | Point prevalence /100,000 (95% CI) | n | Point prevalence /100,000 (95% CI) | n | Point prevalence /100,000 (95% CI) | n | Point prevalence /100,000 (95% CI) | n | Point prevalence /100,000 (95% CI) | n | Point prevalence /100,000 (95% CI) |
| Overall | 93,319 | 23,435  (23,304–23,567) | 103,059 | 25,563  (25,429–25,698) | 25,691 | 25,056  (24,791–25,323) | 28,251 | 27,550  (27,277–27,824) | 89,178 | 25,295  (25,151–25,439) | 98,513 | 27,666  (27,519–27,813) | 106,989 | 21,662  (21,547–21,777) | 116,594 | 23,460  (23,342–23,578) |
| Age |  |  |  |  |  |  |  |  |  |  |  |  |  |  |  |  |
| <=9 | 2,077 | 5,714  (5,478–5,958) | 1,358 | 3,936  (3,733–4,147) | 859 | 8,183  (7,665–8,723) | 573 | 5,779  (5,327–6,256) | 2,186 | 6,660  (6,393–6,935) | 1,492 | 4,740  (4,508–4,981) | 2,953 | 5,832  (5,630–6,040) | 2,107 | 4,334  (4,155–4,519) |
| 10-14 | 2,181 | 9,147  (8,784–9,520) | 1,601 | 7,016  (6,688–7,356) | 842 | 12,992  (12,182–13,835) | 603 | 9,677  (8,954–10,439) | 2,297 | 10,665  (10,256–11,085) | 1,655 | 7,956  (7,591–8,332) | 3,235 | 9,891  (9,570–10,220) | 2,268 | 7,244  (6,959–7,537) |
| 15-19 | 2,017 | 9,126  (8,749–9,513) | 1,879 | 9,062  (8,675–9,461) | 817 | 12,500  (11,707–13,326) | 616 | 10,127  (9,379–10,912) | 2,036 | 10,211  (9,794–10,640) | 1,812 | 9,768  (9,345–10,204) | 2,655 | 9,079  (8,752–9,414) | 2,534 | 9,093  (8,758–9,436) |
| 20-24 | 1,903 | 8,421  (8,063–8,791) | 2,294 | 10,662  (10,253–11,082) | 698 | 11,482  (10,691–12,310) | 726 | 12,755  (11,899–13,649) | 1,968 | 9,693  (9,289–10,108) | 2,578 | 13,389  (12,911–13,878) | 2,188 | 7,777  (7,466–8,096) | 2,757 | 9,896  (9,548–10,253) |
| 25-29 | 2,085 | 8,106  (7,775–8,446) | 3,163 | 12,540  (12,134–12,955) | 711 | 10,596  (9,869–11,357) | 1,015 | 15,390  (14,527–16,284) | 2,171 | 9,822  (9,433–10,222) | 3,202 | 14,754  (14,284–15,233) | 2,426 | 7,629  (7,339–7,926) | 3,586 | 11,522  (11,169–11,882) |
| 30-34 | 2,501 | 9,146  (8,807–9,494) | 3,685 | 13,557  (13,152–13,970) | 786 | 10,597  (9,906–11,320) | 1,059 | 14,766  (13,952–15,608) | 2,471 | 10,401  (10,016–10,796) | 3,705 | 15,523  (15,066–15,989) | 2,775 | 8,201  (7,911–8,498) | 4,132 | 12,188  (11,841–12,541) |
| 35-39 | 3,018 | 10,419  (10,070–10,777) | 4,076 | 14,786  (14,369–15,211) | 872 | 11,544  (10,831–12,286) | 1,143 | 15,871  (15,034–16,735) | 2,937 | 12,012  (11,607–12,426) | 4,096 | 17,160  (16,684–17,645) | 3,479 | 9,911  (9,600–10,228) | 4,811 | 13,297  (12,949–13,652) |
| 40-44 | 3,643 | 13,789  (13,375–14,210) | 4,401 | 17,459  (16,992–17,933) | 1,019 | 14,509  (13,694–15,355) | 1,231 | 18,623  (17,691–19,583) | 3,333 | 14,660  (14,202–15,126) | 4,299 | 20,143  (19,607–20,688) | 4,388 | 12,576  (12,229–12,928) | 5,590 | 16,346  (15,955–16,742) |
| 45-49 | 5,285 | 17,723  (17,291–18,161) | 6,275 | 21,389  (20,921–21,863) | 1,449 | 19,411  (18,519–20,326) | 1,629 | 22,509  (21,551–23,489) | 4,934 | 19,600  (19,112–20,096) | 5,870 | 23,837  (23,306–24,374) | 6,507 | 17,048  (16,672–17,429) | 7,308 | 19,973  (19,565–20,387) |
| 50-54 | 7,317 | 23,320  (22,853–23,792) | 7,752 | 24,798  (24,321–25,281) | 1,987 | 25,852  (24,876–26,847) | 2,208 | 28,912  (27,897–29,943) | 6,595 | 24,895  (24,375–25,421) | 7,014 | 27,291  (26,747–27,840) | 8,645 | 22,464  (22,048–22,885) | 8,760 | 23,685  (23,252–24,121) |
| 55-59 | 8,974 | 30,298  (29,775–30,825) | 8,990 | 30,084  (29,564–30,608) | 2,378 | 32,927  (31,843–34,025) | 2,460 | 34,410  (33,309–35,525) | 7,764 | 31,200  (30,624–31,779) | 7,615 | 31,117  (30,537–31,702) | 9,857 | 28,786  (28,307–29,269) | 9,642 | 29,336  (28,845–29,832) |
| 60-64 | 10,123 | 39,734  (39,132–40,338) | 9,341 | 35,491  (34,913–36,073) | 2,650 | 43,816  (42,561–45,078) | 2,585 | 41,707  (40,476–42,946) | 8,647 | 40,740  (40,078–41,404) | 8,123 | 37,402  (36,758–38,050) | 10,302 | 37,612  (37,038–38,189) | 9,236 | 34,572  (34,002–35,146) |
| 65-69 | 10,747 | 48,182  (47,524–48,840) | 10,009 | 43,370  (42,730–44,013) | 2,741 | 54,116  (52,732–55,496) | 2,518 | 49,141  (47,764–50,520) | 9,688 | 49,243  (48,542–49,944) | 9,136 | 44,257  (43,578–44,938) | 10,941 | 46,935  (46,293–47,578) | 10,219 | 42,436  (41,811–43,063) |
| 70-74 | 11,185 | 58,167  (57,466–58,866) | 10,944 | 52,555  (51,874–53,235) | 2,741 | 64,237  (62,778–65,677) | 2,795 | 60,263  (58,838–61,675) | 11,171 | 58,539  (57,836–59,239) | 10,853 | 52,746  (52,061–53,430) | 12,691 | 56,207  (55,557–56,856) | 12,466 | 51,134  (50,505–51,764) |
| 75-79 | 8,649 | 68,790  (67,972–69,600) | 9,769 | 64,025  (63,258–64,787) | 2,214 | 74,221  (72,611–75,783) | 2,529 | 69,785  (68,260–71,277) | 8,747 | 68,448  (67,634–69,254) | 9,408 | 64,197  (63,414–64,973) | 9,568 | 65,909  (65,131–66,680) | 10,659 | 62,512  (61,781–63,240) |
| 80-84 | 6,500 | 77,556  (76,648–78,446) | 8,464 | 74,103  (73,289–74,904) | 1,624 | 81,444  (79,667–83,129) | 2,082 | 80,293  (78,709–81,809) | 6,749 | 77,352  (76,459–78,227) | 8,192 | 74,304  (73,477–75,118) | 7,733 | 74,686  (73,837–75,521) | 9,620 | 72,719  (71,951–73,477) |
| 85-89 | 3,707 | 84,078  (82,964–85,147) | 5,713 | 81,220  (80,287–82,127) | 927 | 86,152  (83,943–88,162) | 1,562 | 87,556  (85,934–89,053) | 3,777 | 81,489  (80,340–82,597) | 5,750 | 81,077  (80,146–81,983) | 4,581 | 79,587  (78,522–80,621) | 6,638 | 79,269  (78,385–80,133) |
| 90-99 | 1,407 | 84,302  (82,467–86,016) | 3,345 | 83,835  (82,655–84,964) | 376 | 87,442  (83,934–90,423) | 917 | 86,509  (84,304–88,510) | 1,707 | 76,308  (74,489–78,057) | 3,713 | 78,899  (77,705–80,057) | 2,065 | 74,873  (73,210–76,483) | 4,261 | 75,283  (74,137–76,402) |
| School age (5-18) | 5,501 | 8,447  (8,235–8,663) | 4,179 | 6,732  (6,536–6,932) | 2,192 | 11,954  (11,488–12,432) | 1,545 | 8,827  (8,410–9,257) | 5,781 | 9,805  (9,566–10,048) | 4,324 | 7,682  (7,463–7,905) | 7,836 | 8,755  (8,571–8,942) | 6,079 | 7,074  (6,904–7,248) |
| Working age (19-65) | 47,422 | 18,490  (18,340–18,641) | 52,364 | 20,759  (20,601–20,918) | 13,270 | 20,236  (19,929–20,545) | 14,671 | 23,035  (22,709–23,364) | 43,010 | 19,644  (19,478–19,811) | 48,630 | 22,691  (22,514–22,869) | 53,194 | 17,036  (16,905–17,169) | 58,211 | 19,015  (18,876–19,154) |
| <70 years old | 61,871 | 17,580  (17,454–17,706) | 64,824 | 18,810  (18,680–18,941) | 17,809 | 19,403  (19,148–19,661) | 18,366 | 20,672  (20,406–20,940) | 57,027 | 18,691  (18,553–18,830) | 60,597 | 20,333  (20,188–20,478) | 70,351 | 16,064  (15,955–16,173) | 72,950 | 17,033  (16,920–17,146) |
| >=70 years old | 31,448 | 67,980  (67,552–68,405) | 38,235 | 65,328  (64,941–65,713) | 7,882 | 73,321  (72,474–74,155) | 9,885 | 72,159  (71,400–72,908) | 32,151 | 67,745  (67,322–68,165) | 37,916 | 65,312  (64,923–65,699) | 36,638 | 65,467  (65,071–65,861) | 43,644 | 63,535  (63,174–63,895) |

**Supplementary Table 4: At-risk prevalence estimates on 5 March 2019 with and without individuals who left CPRD between 1^st^ January and 5^th^ March 2019**

|  | **Complete follow up time between 1st Jan and 5th March 2019** | | | **Left follow up between 1st Jan and 5th March 2019** | | | **Study population without those that end follow up before 5th March** | | |
| --- | --- | --- | --- | --- | --- | --- | --- | --- | --- |
| **Population** | **n**  **At risk** | **N**  **Total population** | **Prevalence /100,000 (95% CI)** | **n**  **At risk** | **N**  **Total population** | **Prevalence /100,000 (95% CI)** | **n**  **At risk** | **N**  **Total population** | **Prevalence /100,000 (95% CI)** |
| **England** | 223,587 | 990,939 | 22,563  (22,481–22,646) | 2,875 | 11,294 | 25,456  (24,655–26,270) | 220,712 | 979,645 | 22,530  (22,447–22,613) |
| **Northern Ireland** | 53,945 | 205,092 | 26,303  (26,112–26,494) | 443 | 1,192 | 37,164  (34,414–39,979) | 53,502 | 203,900 | 26,239  (26,048–26,431) |
| **Scotland** | 19,6378 | 801,352 | 24,506  (24,412–24,600) | 2,677 | 10,599 | 25,257  (24,432–26,096) | 193,701 | 790,753 | 24,496  (24,401–24,591) |
| **Wales** | 187,695 | 708,670 | 26,486  (26,383–26,588) | 3,260 | 11,067 | 29,457  (28,609–30,316) | 184,435 | 697,603 | 26,438  (26,335–26,542) |
| **Limited to population in England eligible for secondary care data linkage** | | | | | | | | | |
| **Standalone primary care records only** | 175,420 | 744,496 | 23,562  (23,466–23,659) | 2,239 | 8,510 | 26,310  (25,377–27,260) | 173,181 | 735,986 | 23,530  (23,434–23,434) |
| **Linked primary and secondary care records** | 188,716 | 744,496 | 25,348  (25,249–25,447) | 2,455 | 8,510 | 28,848  (27,877–28,824) | 186,261 | 735,986 | 25,308  (25,208–25,407) |

95% CI, 95% confidence interval

**Supplementary Figure 1: Point prevalence of the at-risk group and contributing underlying health conditions by region on 5 March 2014 compared to 5 March 2019** (2019 does not include any individuals in the North East or East Midlands regions; x-axis scales vary by condition)
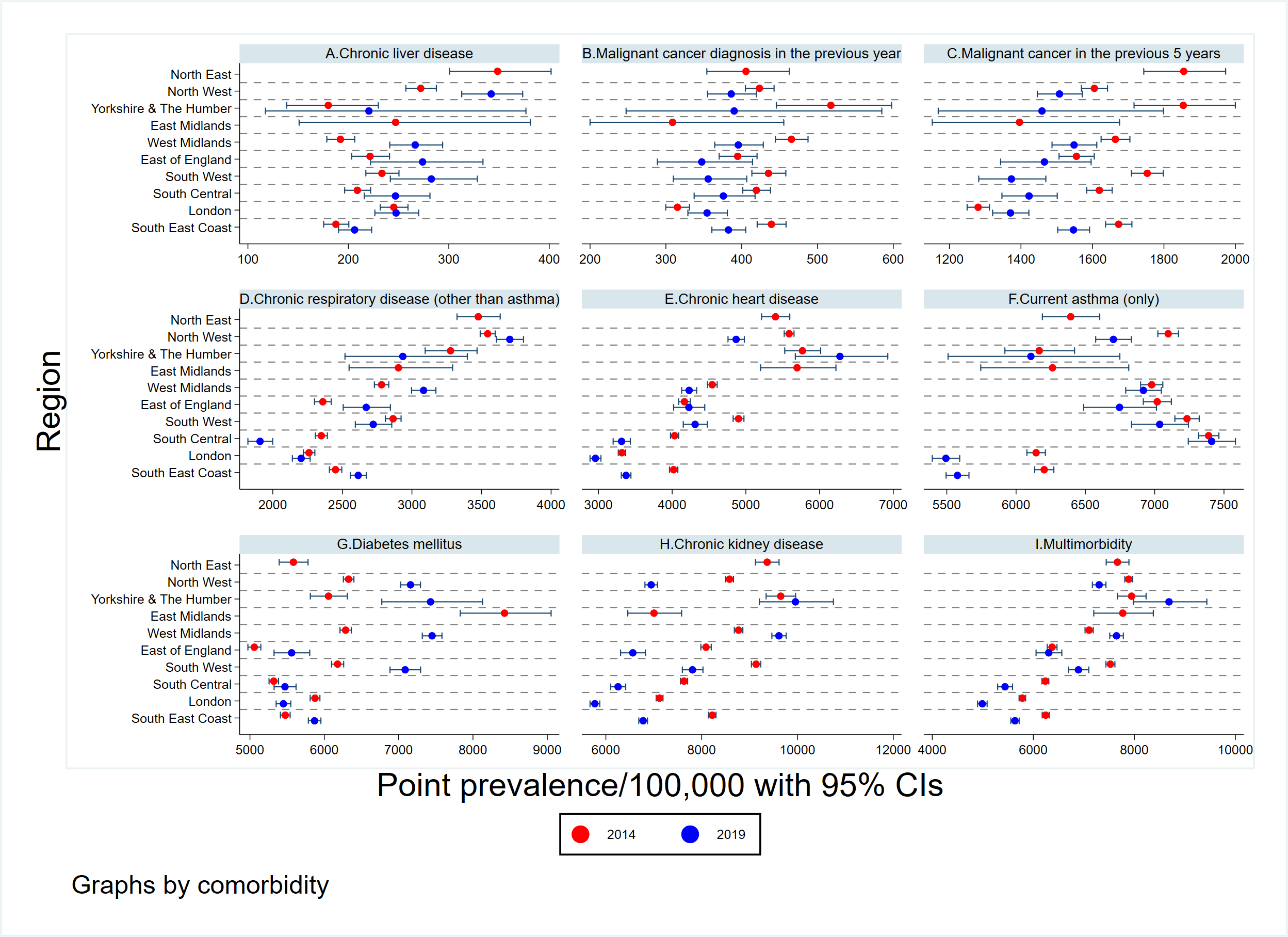

Supplement: Supplementary file 1 — Additional file 1: Supplementary Table 1. Study definitions of underlying health conditions and pregnancy. Supplementary Table 2. Study population characteristics and point prevalence of the COVID-19 at-risk population on 5 March 2014 in the United Kingdom using standalone primary care data, N = 4,730,254. Supplementary Table 3. The at-risk population prevalence on 5 March 2019 stratified by age and sex. Supplementary Table 4. At-risk prevalence estimates on 5 March 2019 with and without individuals who left CPRD between 1st January and 5th March 2019. Supplementary Figure 1. Point prevalence of the at-risk group and contributing underlying health conditions by region on 5 March 2014 compared to 5 March 2019 (2019 does not include any individuals in the North East or East Midlands regions; x-axis scales vary by condition). [file 12889_2021_10427_MOESM1_ESM.docx]
